# Extreme neck elongation evolved despite strong developmental constraints in bizarre Triassic reptiles—implications for neck modularity in archosaurs

**DOI:** 10.1098/rsos.240233

**Published:** 2024-05-15

**Authors:** Adam Rytel, Christine Böhmer, Stephan N. F. Spiekman, Mateusz Tałanda

**Affiliations:** ^1^ Institute of Paleobiology, Polish Academy of Sciences, , Warsaw 00818, Poland; ^2^ Institute of Evolutionary Biology, Faculty of Biology, Biological and Chemical Research Centre, University of Warsaw, , Warsaw 02089, Poland; ^3^ Zoological Institute, Christian-Albrechts-Universität zu Kiel, , Kiel 24118, Germany; ^4^ Staatliches Museum für Naturkunde Stuttgart, Stuttgart 70191, Germany

**Keywords:** Archosauromorpha, geometric morphometrics, *Tanystropheus*, vertebral subregions, ancestral state reconstruction, Tanystropheidae

## Abstract

The Triassic radiation of vertebrates saw the emergence of the modern vertebrate groups, as well as numerous extinct animals exhibiting conspicuous, unique anatomical characteristics. Among these, members of Tanystropheidae (Reptilia: Archosauromorpha) displayed cervical vertebral elongation to an extent unparalleled in any other vertebrate. Tanystropheids were exceptionally ecologically diverse and had a wide spatial and temporal distribution. This may have been related to their neck anatomy, yet its evolution and functional properties remain poorly understood. We used geometric morphometrics to capture the intraspecific variation between the vertebrae comprising the cervical column among early archosauromorphs, to trace the evolutionary history of neck elongation in these animals. Our results show that the cervical series of these reptiles can be divided into modules corresponding to those of extant animals. Tanystropheids achieved neck elongation through somite elongation and a shift between cervical and thoracic regions, without presacral vertebrae count increase—contrary to crown archosaurs. This suggests a peculiar developmental constraint that strongly affected the evolution of tanystropheids. The data obtained just at the base of the archosauromorph phylogenetic tree are crucial for further studies on the modularity of vertebral columns of not only Triassic reptile groups but extant and other extinct animals as well.

## 1. Introduction

**Figure 1.**
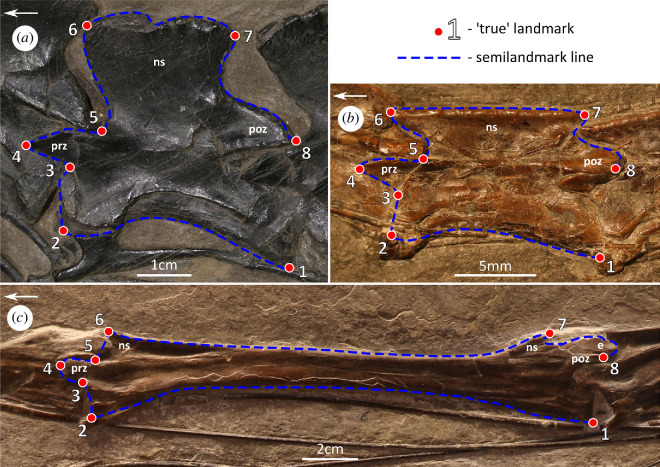
Landmark set used in this study, digitized onto the photographs of (*a*) *Protorosaurus speneri* (WMsN P 47361), (*b*) *Macrocnemus bassanii* (PIMUZ T 4822) and (*c*) *Tanystropheus hydroides* (PIMUZ T 2819). Detailed definitions of the eight true landmarks are provided in the electronic supplementary material. Anatomical abbreviations: e, epipophysis; ns, neural spine; poz, postzygapophysis; prz, prezygapophysis. Arrows indicate anterior direction.

After originating in the Permian, many reptile groups underwent remarkable radiation in the Triassic [1]. This led to the diversification of many modern reptile groups (e.g. archosaurs, turtles and lepidosauromorphs), as well as numerous exceptional animals not closely related to any extant reptiles. *Tanystropheus*, a Middle Triassic archosauromorph, is well known because of its extraordinarily elongated neck, which in some individuals was approximately three times the length of the trunk [2]. This trait has evolved multiple times in many, distantly related groups (e.g. pterosaurs, sauropods, sauropterygians and trachelosaurids) and is not uncommon in itself, but *Tanystropheus* is exceptional when considering how this feature was achieved—it exhibits only 13 cervical vertebrae, most of which are extremely elongated (figure 1*c*) and accompanied by long cervical ribs positioned parallel to the vertebral column [3–6]. For comparison, the closely related *Dinocephalosaurs orientalis* exhibited 33/34 cervical vertebrae [7], while in long-necked sauropterygians, this number could reach over 70 [8]. The morphology of the cervical vertebrae in *Tanystropheus* is unique, owing to their hyperelongation and reduction of the neural spine. They bear some resemblance only to certain pterosaur vertebrae [9]—no modern anatomical analogues exist. Therefore, it is difficult to interpret how this animal functioned and why its vertebral anatomy was modified to such an immense extent. The development and evolution of this feature are poorly known. Many biomechanical interpretations have been suggested [2–4,9–14]; yet, the mystery of the behaviour and habitat of *Tanystropheus* still remain largely unresolved.

Several closely related taxa are grouped with *Tanystropheus* in the family Tanystropheidae, a group of evolutionarily successful Triassic archosauromorphs [15]. Except for *Tanytrachelos* [16], other tanystropheids known from complete and articulated cervical vertebral columns (e.g. *Amotosaurus*, *Macrocnemus*, *Langobardisaurus* and *Ozimek*) possess only eight or nine, significantly shorter (figure 1*b*), cervical vertebrae [17–20]. Members of Tanystropheidae exhibited extraordinarily diverse ecology—some were at least partially aquatic (*Tanytrachelos* and *Tanystropheus*), others lived on land (*Langobardisaurus* and *Macrocnemus*) and *Ozimek* was interpreted as a possible glider [2,3,16,18,19]. This multiplicity of forms can potentially be linked to the distinctive variance of size, morphology and count of tanystropheid cervical vertebrae. However, we have little understanding of how this diversity has evolved within this clade and how it contributed to the evolutionary success of the group. A new, abundant tanystropheid material from Miedary (Upper Silesia, southern Poland) can potentially give us insight into this subject [21]. Most of the well preserved tanystropheid specimens from around the world are diagenetically flattened and can therefore only be assessed unilaterally. In contrast, the *Tanystropheus* specimens from Miedary are three-dimensionally preserved, allowing for observation of much more detail.

Understanding how different developmental processes interact to generate variation in anatomical features is crucial in studies on phenotypic evolution. The adult morphology of the vertebral column directly reflects the mechanisms that generate vertebral counts (somitogenesis) and their regionalization (homeotic effects) during embryonic development [22–26]. Many morphometric studies on the modularity of the vertebral column in extant and extinct taxa have been conducted in recent years (e.g. [27–37]). A new method of morphological subregion (i.e. module) differentiation in the vertebral column of archosaurs has been proposed by Böhmer *et al*. [28] and later used on different animal lineages [30,38]. This method is conducted by determining morphologically distinguishable modules of cervical vertebrae and comparing them with expression boundaries of the Hox genes to establish a developmental correlation between them. These studies have shown that integration of genes and morphology derived from both extant and extinct taxa can give us an extensive insight into the regionalization of the neck and thus the evolution and development of specific cervical characteristics.

In the past, only some basic morphometric methods have been used to evaluate tanystropheid fossils [3,4,9,11,12]. Herein, we use more sophisticated, two- and three-dimensional geometric morphometric methods (GMMs) to investigate the shape variation in the postaxial cervical vertebrae within Tanystropheidae and other closely related archosauromorphs, to differentiate the morphological subregions in the necks of the studied taxa and to compare them with the results available for both extant and extinct animals. Additionally, we used ancestral state reconstruction to analyse cervical and dorsal vertebral count changes across the phylogenetic tree of early archosauromorphs. With these data, we trace the evolution of the tanystropheid cervical column in its transition from relatively short-necked non-tanystropheid archosauromorphs to highly specialized forms like *Tanystropheus*.

## 2. Material and methods

We investigated the morphological variation within the dataset with two-dimensional GMM. Only postaxial cervical morphology has been studied—atlas and axis have been excluded from the dataset owing to their highly modified morphology, poor preservation and difficulties in finding homologous landmarks shared with the more caudally located vertebrae. Over 100 cervical vertebrae (both isolated and those from articulated cervical columns) have been analysed. These included vertebrae of the tanystropheids *Tanystropheus* (*T*. ‘*conspicuus*’, *T. hydroides* and *T. longobardicus*) and *Macrocnemus* (*M. bassanii* and *M. fuyuanensis*), as well as the two other early archosauromorphs [15] *Prolacerta broomi* and *Protorosaurus speneri*. Owing to the aim of the study, only species with completely preserved cervical columns were included. Incomplete preservation precluded us from incorporating other tanystropheid genera into the data matrix. A detailed list of the analysed specimens is provided in the electronic supplementary material.

The landmark set used in the GMM analysis (figure 1) was created to capture the shape of the vertebrae within the cervical columns of the analysed taxa. It consisted of 8 true landmarks and 7 semilandmark lines encompassing 73 semilandmarks. True landmarks used in this study can be classified as type II landmarks *sensu* Bookstein [39]. Semilandmarks were digitized between the true landmarks on the outline of the vertebrae. A number of semilandmarks encompassed by each specific semilandmark line were designated to cover the shape of the local contour of the bone in detail. These values depended mostly on the specific shape variance extremes, exhibited by some of the taxa—the number of semilandmarks was chosen to properly outline the most complex curvature within the dataset.

Owing to incomplete preservation and preparation, no specimen of *Prolacerta broomi* could provide us with the full morphological data of the whole cervical series of a single individual. To overcome this issue, a slightly adjusted landmark set has additionally been used to investigate its morphological variance. Further details and results of this analysis are provided in the electronic supplementary material, while the results recovered from the main dataset for the nearly complete specimen BP/1/2675 are contained within the following sections.

We collected the morphological data using the tps software package [40]. After creating a *.tps file using the utility software tpsUtil v1.78, the landmarks and semilandmark lines were digitized onto the high-quality photographs of the vertebrae in the tpsDig v2.31 software. The semilandmark lines were resampled, relocating the semilandmarks so that the associated line is made out of sections of the same length (with semi-random placement of the landmarks along the line).

The data we acquired consisted of *x*/*y* coordinates of each of the landmarks digitized for each of the vertebrae. The data were subsequently superimposed using general Procrustes analysis in Past v4.15 [41]. We then performed a principal component analysis (PCA) on the transformed data. To visualize morphological variance in the dataset, we created thin-plate splines with the tpsRelw v1.70 software. This program uses the relative warps (RW) method to analyse the morphological variance in a given dataset. To help identify morphological subregions, we performed non-hierarchical *K*-means analysis in Past v4.15 [41], with the *K* value based on the elbow method and the average silhouette method. The results of the PCA, RW, *K*-means and cluster analysis served as a base for subregion differentiation within the cervical series of the studied taxa. We established clusters on the basis of shape similarity between the individual vertebrae, comparing the results obtained with all of the methods listed above.

To check the potential significance of using only the two-dimensional data for the results of the study, we conducted an additional comparative analysis. Some of the three-dimensionally preserved specimens were surface scanned. Landmarks were digitized onto the created models in the Landmark v3.0.0.6 software [42]. Then, we compared the created dataset to the results obtained with two-dimensional GMM analysis, following the methodology proposed by Cardini [43]. Thus, the potential differences and data loss related to exclusion of the third dimension could be assessed. The results of the dataset comparison, as well as additional methodological remarks, institutional abbreviations and details concerning the data acquisition, are provided in the electronic supplementary material. Additionally, we performed an ancestral state reconstruction to analyse presacral vertebral count changes across the studied taxa. We used a maximum parsimony approach in Mesquite v3.51 [44]. We analysed two characters, namely the cervical and dorsal vertebral counts, which were treated as ordered. We used taxa from Spiekman *et al*. [15] and the strict consensus tree of the ‘analysis four’ contained therein.

The data gathered by conducting the PCA, RW, *K*-means and cluster analyses were based on the most completely preserved cervical series for all examined taxa, except *Tanystropheus* spp. For the latter, all vertebrae available for the studies were analysed as one subset, owing to their relatively large number and non-existence of any described *Tanystropheus* individual in which all postaxial cervical vertebrae are preserved sufficiently to be considered for our study. Even though our analyses covered all of the best preserved published specimens known for the studied taxa, the dataset was limited, owing to the nature of the fossil record for this group. This precluded us from analysing intercolumnar disparity, which has been a subject of recent studies on the subject [45–47].

## 3. Results

The results of the analysis comparing the two- and three-dimensional data for the same specimens are provided in the electronic supplementary material. They support the viability of using the two-dimensionally preserved specimens as a proxy for the morphological variation comparison, because the exclusion of the third dimension did not affect the final data layout in a significant way.

**Figure 2.**
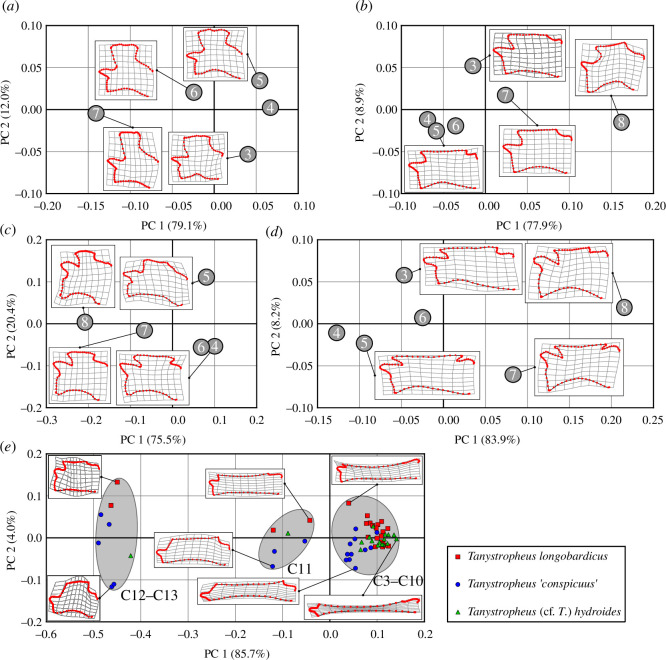
Relative warps analysis results. The plots present shape variation within the cervical columns of the investigated taxa: (*a*) *Protorosaurus speneri* (WMsN P 47361), (*b*) *Macrocnemus bassanii* (MSNM BES SC 111), (*c*) *Prolacerta broomi* (BP/1/2675), (*d*) *Macrocnemus fuyuanensis* (GMPKU-P-3001) and (*e*) *Tanystropheus* spp. (multiple specimens, for the full list refer to the electronic supplementary material). Zero point represents the average (consensus) shape of the vertebrae within each dataset. Numbers within the graphs indicate the position of the vertebrae within cervical columns. The ellipses within (*e*) contain the three main vertebrae clusters observed in the morphospace (CVs3–10; CV11; CVs12–13).

RWs analysis results (figure 2) illustrate the morphological variation within the analysed dataset. The relative elongation of the vertebrae is the prevalent element influencing the value score calculated for each cervical vertebra along principal component 1 (PC1) axes. In all of the analysed taxa, PC1 explained a very large portion of the variance (75.5–85.7%). In *Macrocnemus bassanii*, *Macrocnemus fuyuanensis*, *Prolacerta broomi* and *Protorosaurus speneri*, the cervical vertebrae cluster in a similar way, with the anterior three or four (depending on the species) cervical vertebrae clustering together in the morphospace, and the following two, less elongated vertebrae each occupying a distinct position in relation to the other elements of the same cervical column. In *Tanystropheus* spp., a similar pattern can be observed, with three clusters that are well defined in the morphospace. The first cluster is composed of the similarly elongated anterior vertebrae (CV3–10), the second cluster includes the eleventh cervical vertebra (CV11) and the third one is composed of the last, relatively short, cervical vertebrae (CV12–13). This clustering pattern, in which the two or three posteriormost cervical vertebrae constitute two distinct subsets, can also be observed within the results of the PCA, cluster (see electronic supplementary material) and *K*-means analyses. For the latter, the used algorithms suggested the optimal number of clusters to be two or three, which is congruent with the findings of previous studies [28,38]. With *K* = 2, one or two posteriormost cervical vertebrae were assigned to a distinct subset in all of the studied taxa. With *K* = 3, in *Protorosaurus speneri*, *Prolacerta broomi* and *Macrocnemus* spp., each of the two posteriormost cervical vertebrae clustered separately from the preceding vertebrae, while in *Tanystropheus* spp., the two posteriormost cervical vertebrae clustered together, while the CVs11 constituted their own subset. This mirrors the results of the other analyses conducted in this study.

The ancestral state reconstruction allowed us to analyse the presacral vertebral count changes across the studied taxa and their relatives. For Archosauromorpha, 25 or 26 presacral vertebrae (seven cervical, 18–19 dorsal) were recovered as ancestral, which is well represented in *Protorosaurus speneri*. Correspondingly, 25 (eight cervical, 17 dorsal) presacral vertebrae characterized the ancestor of Tanystropheidae, as observed in, for example, *Macrocnemus* spp. Among tanystropheids, this formula can be confidently declared as not present only in *Tanystropheus* spp., *Tanytrachelos ahynis* and their ancestors. In these taxa, the dorsal/cervical vertebral count ratio is lower, while they retain the ancestral count of 25 presacral vertebrae. Therefore, the observed increase in the number of cervical vertebrae was achieved through ‘cervicalization’ of some of the dorsals. Detailed results of the ancestral state reconstruction are presented in the electronic supplementary material.

## 4. Discussion

### 4.1. Ancestral vertebral counts and cervical column subregions in Archosauromorpha

**Figure 3.**
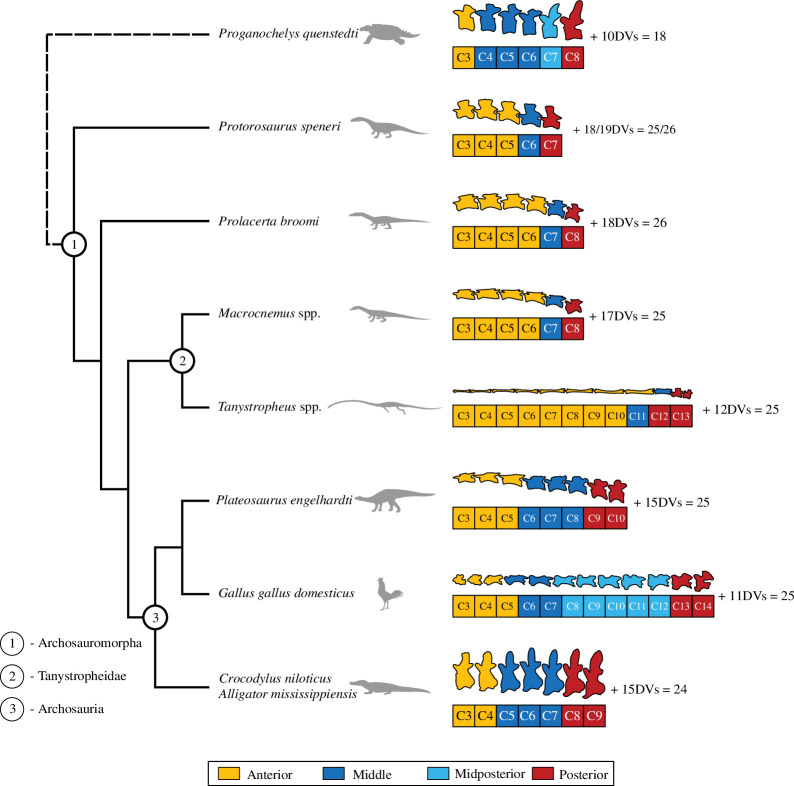
Phylogenetic distribution of modules in the vertebral columns of the taxa studied herein, as well as a stem-turtle *Proganochelys quenstedti* [30] and archosaurs [28] analysed in other studies. Tree topology after Spiekman *et al*. [15]. Colours indicate correspondence to a specific module. Equations to the right of the cervical series include the number of dorsal vertebrae and the total number of presacral vertebrae in each of the taxa. Animal body outlines, and their vertebrae are not to scale.

Although the cervical vertebrae of the analysed taxa have high variable morphologies (see figure 1), a similar clustering arrangement can be observed between cervical vertebrae of all of the studied taxa. Therefore, the obtained results suggest the existence of analogous morphological subregions in the vertebral column of the genera analysed herein (figure 3). As *Protorosaurus speneri* is considered one of the basal-most archosauromorphs [15,48–50], if not a sister taxon to all others of them, the morphological modularity pattern identified in this taxon might therefore be closely related to the ancestral condition of Archosauromorpha. Although *Protorosaurus speneri* possessed seven cervical and 18 or 19 dorsal vertebrae (A.R., personal observation) [23,51,52], some authors have hypothesized that the ancestral state for vertebral counts in Archosauromorpha included a significantly higher number of presacral vertebrae—around 31 [23]. This reconstruction is highly unlikely, especially in the light of new studies on the phylogeny of the group [15,50]. Our analysis of the ancestral state of this clade indicates only 25 or 26 presacral vertebrae (see electronic supplementary material). This is in accordance with the predominantly constant presacral vertebral count in non-archosauriform archosauromorphs (e.g. most tanystropheids, rhynchosaurs, *Protorosaurus speneri* and *Prolacerta broomi*) as well as the early archosauriforms (see [23] and electronic supplementary material therein), with *Protorosaurus speneri* serving as the model example of this condition. It is, however, worth noting that considerably more presacral vertebrae are present in the recently recognized family Trachelosauridae, e.g. *Dinocephalosaurus orientalis* and *Trachelosaurus fischeri* [7,53–55].

*Protorosaurus speneri*, *Prolacerta broomi* and *Macrocnemus* spp. exhibit a strikingly similar layout of the proposed morphological subregions—three or four anterior, one middle and one posterior cervical vertebrae can be differentiated (figure 3). Both in *Prolacerta broomi* and *Macrocnemus* spp., one cervical vertebra has been added to the anterior subregion. This evolutionary change was already briefly suggested in the literature [3]. Although both of these genera exhibit an identical modularity pattern, it may have been achieved convergently, as the vertebral column of *Prolacerta broomi* contains one more presacral (dorsal) vertebra than those of *Macrocnemus* spp. (respectively, 26 and 25 [20,56–58]). The exact temporal configuration of changes in somitogenesis and homeotic shifts for these genera cannot be determined on the basis of the data available.

### 4.2. Cervical column subregions in *Tanystropheus* and their evolution

The layout of morphological subregions in the neck of *Tanystropheus* spp. can be assessed in detail based on our results (figure 3). Similar to the other studied genera, as well as some of the archosaurs analysed in a previous study [28], three possible postaxial subregions in the neck of *Tanystropheus* spp. can be recognized, on the basis of the well-defined morphological clusters of vertebrae (figure 2). The anterior subregion is composed of eight very elongated vertebrae, which are succeeded by a transitional eleventh vertebra that constitutes the middle subregion. The last two, relatively short, cervical vertebrae are contained within the posterior subregion.

If we consider *Protorosaurus speneri* as an outgroup for all other archosauromorphs, with five postaxial cervical vertebrae divided into three subregions, and using parsimony, we can recreate a hypothetical history of the specialized neck anatomy evolution in tanystropheids and some of their close archosauromorph relatives. In *Tanystropheus* the anterior subregion has been expanded by five vertebrae and the posterior subregion by one. Some of these changes might have already been present in the ancestors of *Tanystropheus*, yet the majority of other tanystropheids exhibit only eight or nine cervical vertebrae [17–20,57], with *Tanytrachelos ahynis* and *Tanystropheus* spp. being the notable exceptions [5,16,59]. The intermediate stages of evolution remain unknown. It has been proposed that the homeotic shift observed in *Macrocnemus bassanii* was inherited by *Tanystropheus* [3]. In fact, as no tanystropheid with less than eight cervical vertebrae is currently known, the transition from seven to at least eight cervical vertebrae, with no change in the presacral vertebral count, evolved on the stem to Tanystropheidae, or earlier in their archosauromorph ancestors. The layout of morphological subregions in *Macrocnemus* spp. is very similar to that of *Tanystropheus* and can indicate that the anterior subregion was expanded first in the evolutionary history of neck elongation in Tanystropheidae.

### 4.3. Neck elongation in *Tanystropheus* compared with other vertebrates

While neck elongation is always achieved through a combination of the same three developmental mechanisms (somitogenesis, homeotic shifts and somite growth), the asymmetrical intensity of expression of each of these processes throughout the evolution of different groups results in distinctly different cervical morphologies [23,25,26,60,61], which can be easily observed taking into consideration several well-known examples. The presacral and cervical counts of mammals are highly conserved owing to well-documented developmental constraints [60,62,63], thus long necks of extant giraffids are a product of cervical vertebrae elongation only [64]. On the other hand, the cervical characteristics of many of the long-necked archosaurs (e.g. birds and sauropods) are a result of simultaneous action of accelerated somitogenesis, homeotic changes and somite growth [23,28]. Contrary to that, in many eusauropterygian lineages, the individual vertebrae were predominantly short, but their number was significantly higher than in other vertebrates, thus increasing the vertebral column length, with homeotic effects also partaking in the process [25,60]. Authors of an extensive case study conducted on Sauropterygia [25] found no taxon in which only homeotic shifts resulted in neck elongation; they were always accompanied by changes in the presacral vertebral count. In this respect, we observe that the cervical anatomy of *Tanystropheus* serves as an extreme example of substantial neck elongation achieved by extensive ‘cervicalization’ of dorsal vertebrae and elongation of individual cervical vertebrae, as evidenced by the results of the ancestral state reconstruction, but without any evidence of prolonged somitogenesis. These characteristics have been briefly noted in different contexts by previous authors [3,11,12,23,60,65]. Yet, herein, we note for the first time that no tanystropheid with a presacral vertebral count of more than 25 has been described [5,15,17–20,57–59]. *Tanytrachelos ahynis* was mentioned as having 13 dorsal, and 12 [59] or 13 [16] cervical vertebrae, but the latter character is difficult to prove owing to imperfect preservation of the specimens assigned to this genus. Thus, potentially all tanystropheids may exhibit a similar pattern of neck evolution—lacking any signs of accelerated somitogenesis, but with intensive cervical elongation and (in some genera) homeotic effects coacting to result in a relative length increase of the cervical column. Interestingly, the non-archosauriform archosauromorph *Dinocephalosaurus orientalis* exhibits approximately 62 presacral vertebrae (33–34 cervical and 28–29 dorsal vertebrae [7])—a result of prolonged somitogenesis. Other (putative) trachelosaurids, *Trachelosaurus fischeri* and most likely *Gracilicollum latens* and *Austronaga minuta*, also exhibit remarkably high vertebral counts [53,66,67]. As in trachelosaurids, it could be expected that with the inferred change of habitat from terrestrial to marine in the tanystropheid ancestors of *Tanystropheus* [2,4,13], which is a drastic transition uncommon in archosauromorphs, the vertebral formula would also change, with additional presacral vertebrae being added. Similar innovations have appeared convergently during the evolution of further adaptations to the aquatic lifestyle in other reptile groups as well—choristoderans, mesosaurs, sauropterygians and thalattosaurs [23–25,60,68]. This is not the case in *Tanystropheus*, or in fact any other tanystropheid, as we do not see an increase of the presacral vertebral count in any of them. It would seem that the vertebral formula plasticity seen in other reptile groups may have been significantly constrained in Tanystropheidae. This factor, combined with presumed selective pressure promoting neck elongation, connected with the environmental transition, produced the unique anatomy of *Tanystropheus*.

The major differences in cervical anatomy characteristics between *Tanystropheus* and other reptiles are further underlined by the results of this study (figure 3). Based on the available data, including the insights provided herein, and using phylogenetic bracketing [69], it can be noted that throughout the evolution of archosaurs, possibly only the middle and posterior modules of their necks were expanded, with birds exhibiting an additional midposterior module [28]. While in *Tanystropheus* the posterior module was also expanded by one vertebra, the middle module remained conserved to only one vertebra while five vertebrae were incorporated into the anterior module. In all of the studied tanystropheid vertebrae, the anterior cervical vertebrae are always the most elongated. Following this observation, it may be noted that the predominant expansion of the anterior module of the neck of *Tanystropheus* resulted in the maximized relative elongation of the neck. Thus, it can be hypothesized that the unique cervical morphology exhibited by *Tanystropheus* is a product of strong selective pressure affecting the evolution of this animal towards neck elongation, which was achieved through extreme somite growth and homeotic shifts, owing to conserved or constrained presacral vertebral count. Interestingly, a similar pattern of the relative neck length increase can be observed in some pterosaur lineages, in which the presacral vertebrae count was mostly conserved, or even decreased through their evolution [23]. Some of them exhibit a cervical vertebral morphology superficially analogous to those of tanystropheids [9]. This comparison proves that under similar developmental and evolutionary conditions, fairly similar vertebral morphology can be convergently achieved by distantly related animals, despite clear and extensive differences in their ecology. Nonetheless, the bauplan of *Tanystropheus* stands out even among animals in which the neck elongation evolved without presacral count increase. The combination of the high extent of the dorsal vertebrae cervicalization and the extreme elongation of cervical vertebrae and ribs is unparalleled in any other taxa—no close analogues are known.

## 5. Conclusion

Our research demonstrates that certain uniform, quantifiable morphological patterns can be used to trace the evolution of a trait and its development. Moreover, this study provides additional support for the viability of using two-dimensionally preserved skeletons for assessing shape variability with GMMs. On the basis of the results of the GMM analyses performed herein, the modularity patterns in the necks of early archosauromorphs *Protorosaurus speneri*, *Prolacerta broomi*, and several tanystropheids, were established, which allows for tracing the evolution of their vertebral columns. The modularity pattern observed in the cervical vertebrae of early archosauromorphs is an important basis for future broader research on the vertebral evolution of vertebrates. Neck elongation in *Tanystropheus* spp. occurred in consequence of the elongation of vertebrae and incorporation of dorsal vertebrae into the neck, without accelerated somitogenesis partaking in the process, and with the anterior morphological subregion being predominantly expanded. This particular case of neck elongation constitutes a good example of morphological evolution not only under strong natural selection but also under strong developmental constraints, reminiscent of the conservative mammalian cervical count. These trade-offs bring a different perspective to the discussions around adaptation and function of the *Tanystropheus* neck and its role in the enigmatic mode of life of this animal. The insights provided herein further highlight the uniqueness of cervical anatomy of *Tanystropheus* among vertebrates, not only on the grounds of pure morphology, but evolutionary and developmental aspects as well.

## Data Availability

Most of the data are contained within the supplementary material [71]. All of the untransformed morphometric data used in this study can be accessed by following the link to the Dryad repository: https://doi.org/10.5061/dryad.bvq83bkfx [70]. The .dat files can be opened with the free PAST software (https://past.en.lo4d.com/windows).
